# Factors associated with healthcare seeking for childhood illnesses among mothers of children under five in Chad

**DOI:** 10.1371/journal.pone.0254885

**Published:** 2021-08-05

**Authors:** Eugene Budu, Abdul-Aziz Seidu, Edward Kwabena Ameyaw, Ebenezer Agbaglo, Collins Adu, Felicia Commey, Kwamena Sekyi Dickson, Kenneth Setorwu Adde, Bright Opoku Ahinkorah

**Affiliations:** 1 Department of Population and Health, University of Cape Coast, Cape Coast, Ghana; 2 College of Public Health, Medical and Veterinary Sciences, James Cook University, Townsville, Queensland, Australia; 3 School of Public Health, Faculty of Health, University of Technology Sydney, Sydney, Australia; 4 Department of English, University of Cape Coast, Cape Coast, Ghana; 5 Department of Health Promotion and Disability Studies, Kwame Nkrumah University of Science and Technology, Kumasi, Ghana; Helen Keller International, SIERRA LEONE

## Abstract

**Background:**

Poor healthcare-seeking behaviour is a major contributing factor for increased morbidity and mortality among children in low- and middle-income countries. This study assessed the individual and community level factors associated with healthcare-seeking behaviour for childhood illnesses among mothers of children under five in Chad.

**Methods:**

The study utilized data from the 2014–2015 Chad Demographic and Health Survey. A total of 5,693 mothers who reported that their children under five had either fever accompanied by cough or diarrhea or both within the two weeks preceding the survey were included in this study. The outcome variable for the study was healthcare-seeking behaviour for childhood illnesses. The data were analyzed using Stata version 14.2. Multilevel binary logistic regression model was employed due to the hierarchical nature of the dataset. Results were presented as adjusted odds ratios (aOR) at 95% confidence interval (CI).

**Results:**

Out of the 5,693 mothers who reported that their children under five had either fever accompanied by cough, diarrhea or both at any time in the 2 weeks preceding the survey, 79.6% recalled having sought treatment for their children’s illnesses. In terms of the individual level factors, mothers who faced financial barriers to healthcare access were less likely to seek healthcare for childhood illnesses, relative to those who faced no financial barrier (aOR = 0.80, 95% CI = 0.65–0.99). Mothers who reported that distance to the health facility was a barrier were less likely to seek healthcare for childhood illnesses, compared to those who faced no geographical barrier to healthcare access (aOR = 79, 95% CI = 0.65–0.95). Mothers who were cohabiting were less likely to seek healthcare for childhood illnesses compared to married mothers (aOR = 0.62 95% CI = 0.47–0.83). Lower odds of healthcare seeking for childhood illnesses was noted among mothers who did not listen to radio at all, relative to those who listened to radio at least once a week (aOR = 0.71, 95% CI = 0.55–0.91). Mothers who mentioned that their children were larger than average size at birth had a lesser likelihood of seeking childhood healthcare, compared to those whose children were of average size (aOR = 0.79, 95% CI = 0.66–0.95). We further noted that with the community level factors, mothers who lived in communities with medium literacy level were less likely to seek childhood healthcare than those in communities with high literacy (aOR = 0.73, 95% CI = 0.53–0.99).

**Conclusion:**

The study revealed that both individual (financial barriers to healthcare access, geographical barriers to healthcare access, marital status, frequency of listening to radio and size of children at birth) and community level factors (community level literacy) are associated with healthcare-seeking behaviour for childhood illnesses in Chad. The government of Chad, through multi-sectoral partnership, should strengthen health systems by removing financial and geographical barriers to healthcare access. Moreover, the government should create favourable conditions to improve the status of mothers and foster their overall socio-economic wellbeing and literacy through employment and education. Other interventions should include community sensitization of cohabiting mothers and mothers with children whose size at birth is large to seek healthcare for their children when they are ill. This can be done using radio as means of information dissemination.

## Background

Healthcare seeking for childhood illnesses is an important measure of the level of welfare of communities [1]. Vulnerable populations across the globe, including children, are subjected to poorer health outcomes [2]. Globally, about 6.2 million children died in 2018, and 5.3 million of these deaths were among children under the age of five years [3, 4]. Sub-Saharan Africa remains the region with the highest child mortality and under-5 mortality rates in the world, with 1 child in 13 dying before his or her fifth birthday [3, 4]. Under-five mortality rates in Chad declined from 189 per 1,000 live births 1999 to 119 deaths per 1,000 live births in 2018 [5]. However, this rate is still high.

The global community has garnered efforts to end preventable deaths of children, with the setting of several goals and initiatives such as the Sustainable Development Goals (SDGs) and the United Nations Global Strategy for Women’s, Children’s, and Adolescent’s Health (2016–2030). Preventable diseases such as malaria, pneumonia, preterm birth complications, and diarrhea are the common causes of childhood deaths globally [5].

Access to healthcare contributes significantly to reduction in child mortality [6]. In low- and middle-income countries (LMICs), common causes of under-five mortality and morbidity can be reduced considerably with prompt healthcare seeking behaviour for childhood illnesses [7]. However, a large number of caregivers of sick children in LMICs do not visit health centers [6], due to a number of factors which include delays in the provision of medical care by healthcare staff, lack of financial support, distance to the health facilities and illiteracy [6, 8, 9].

Healthcare in Chad is provided through direct payment, free access to selected services, health insurance, and health mutual (payment of cost of healthcare by both private and public health organization) [10]. More than half of the total health expenditure is through out-of-pocket payment, with free access to some selected healthcare through the financial support of the state. Health insurance coverage in Chad is less than 2% and often controlled by large corporations for the benefit of their employees while health mutual is currently in its pilot phase in the southern regions [10].

Previous studies have shown that parent’s socio-cultural characteristics play a great role in the decision to seek medical care for their children’s illnesses [8, 11–13]. In LMICs, several studies have shown that parents, notably mothers, usually have little knowledge of appropriate medical treatment for their children’s ailments [8, 11]. Several studies observed that some mothers in LMICs opt for traditional treatment ahead of modern healthcare services for their children’s diseases often due to their availability at the local level [8, 11, 13]. Previous studies identified that some common factors such as child’s age child sex, mothers’ educational level, place of residence, distance to the nearest health center, and family’s socio-economic status as barriers to healthcare access and healthcare seeking for childhood illnesses [11–20].

Based on the child health situations in Chad, a better understanding of factors associated with healthcare seeking for childhood illnesses among mothers in the country is necessary. The aim of this study was to examine the factors associated with healthcare seeking for childhood illnesses among mothers in Chad. Findings from the study will help government and non-governmental organisations in Chad to implement policies and programmes aimed at improving child health and survival.

## Methods

### Study design

This study was a cross-sectional study that utilized data from the 2014–15 Chad Demographic and Health Survey (CDHS), which is the most recent DHS conducted in the country. The CDHS is conducted by the National Institute of Statistics, Economic and Demographic Studies (INSEED) and the Inner-City Fund (ICF) International [21].

### Population and sampling

The CDHS utilized a stratified sampling design to recruit eligible participants. The national territory was demarcated into twenty-one study areas with reference to the 22 regions and the city of N’Djaména. Two strata were created in each field (urban and rural). In all, 626 primary survey units (PSUs) or clusters were systematically selected from the list of enumeration areas that were predefined during the 2009 General Population and Housing Census. Households in each cluster constituted the list from which eligible households were selected, with 25 households per cluster in the urban locations and 30 households per cluster in rural locations at random. A total of 17, 965 households from 4,075 urban areas in 163 clusters and 13,890 rural households nested in 463 clusters were selected. All resident mothers 15–49 years or those present the night preceding the survey were eligible to be interviewed. A total of 5,693 mothers reported that their children under five had either fever accompanied by cough or diarrhea or both within the two weeks preceding the survey. This constituted the sample size for our study.

### Definition of variables

#### Outcome variable

The outcome variable for the study was healthcare seeking behaviour for childhood illnesses. This variable was derived as a composite variable from two questions: “Did [NAME] receive treatment for diarrhea?” and “Did [NAME] receive treatment for fever accompanied by cough?” The responses were “Yes” and “No” in the CDHS. All mothers who responded “Yes” to either of the two questions were considered as seeking healthcare for childhood illnesses (coded as 1) whilst those who did not seek healthcare for any of the two childhood illnesses were coded as 0.

#### Independent variables

There were 21 independent variables made up of 18 individual level variables and three community level variables. None of these variables was selected a prior; instead, the selection was based on conclusions drawn by earlier studies on healthcare seeking for childhood illnesses as well as their conceptual and theoretical bearing on healthcare seeking for childhood illnesses [22, 23].

### Individual level variables

The individual level variables were difficulty with distance to the facility, difficulty in getting money for treatment, difficulty with getting permission to visit a health facility, and difficulty in not wanting to go for medical help alone (each was coded as big problem and not a big problem). These related to geographical, financial, and partner support barriers faced by mothers when accessing healthcare. Big problem means the respondents considered each of these as a barrier to healthcare access whiles not a big problem means that they were not considered as barriers. Other individual level variables were mothers’ age (15–19, 20–24, 25–29, 30–34, 35–39, 40–44, and 45–49), marital status (married and cohabiting), healthcare decision-making capacity (alone and not alone), parity (one birth, two, three, and four or more), employment status (working or not working), religion (Christianity, Islam, and no religion), frequency of exposure to media (reading newspaper, listening to radio, watching television) which were coded as not at all, less than once a week, and at least once a week, sex of household head (male and female), mother’s subjective perception of the size of child at birth (less than average, average, or smaller than average), birth order (one, two to four, and five and above births), twin status (single or multiple births), and sex of child (male and female).

### Community level variables

Community literacy level (categorized into low, medium, and high), community socio-economic status (captured as low, medium, and high), and residence (rural and urban) were the community level variables. The categorisation of community literacy level and community socio-economic status into low, medium and high was not directly available in the data but generated from maternal education and household wealth quintile through a method of aggregation at the cluster level.

### Statistical analyses

We employed both descriptive and inferential analytical approaches. First, we computed the proportion of mothers who sought healthcare for childhood illnesses across the individual and community level variables. Next, a Chi-square test was carried out to assess the level of significance between the independent variables and healthcare seeking for childhood illnesses (see Table 1). At the bivariate analysis stage, due to multiple-comparisons, we introduced a correction method by using the Bonferroni correction method [24]. This was done by dividing the alpha rate (p = 0.05) by the number of analysis performed (21 explanatory variables) [25, 26], that is, 0.05/21 = 0.002. Therefore, at the bivariate analysis, statistical significance was declared at p≤0.002. Following the hierarchical nature of the dataset, the multilevel logistic regression model (MLRM) was employed after the bivariate analysis to examine the predictors of healthcare seeking for childhood illnesses. This comprises fixed effects and random effects [27]. The fixed effects of the model were gauged with binary logistic regression, which resulted in adjusted odds ratios (aORs) (see Table 2). The random effects, on the other hand, were assessed with intra-cluster correlation (ICC) [28] (see Table 2). The sample weight (v005/1,000,000) was applied in all the analyses to control for over- and under-sampling. All the analyses were carried out using Stata version 14.2.

**Table 1:** Healthcare seeking for childhood illnesses across independent variables.

Variables	Weighted N	Weighted %	Health seeking behavior (%)	χ2 (p-value)
			79.6	
**Getting permission for medical care for self**				0.2 (0.692)
Big problem	2539	44.6	80.3	
Not a big problem	3154	55.4	79.1	
**Getting money for medical care for self**				18.2 (<0.001)
Big problem	4455	78.3	78.7	
Not a big problem	1238	21.7	82.9	
**Distance to facility for medical care for self**				16.8 (<0.001)
Big problem	3806	66.9	78.4	
Not a big problem	1887	33.1	82.2	
**Wanting to go for medical care alone**				5.7 (0.017)
Big problem	2522	44.3	78.4	
Not a big problem	3171	55.7	80.6	
**Community literacy level**				17.4 (<0.001)
Low	2247	39.5	79.8	
Medium	1189	20.9	74.7	
High	2257	39.6	82.0	
**Community socio-economic status**				24.4 (<0.001)
Low	3652	64.1	78.0	
Medium	370	6.5	77.2	
High	1671	23.4	83.7	
**Residence**				2.4 (0.125)
Urban	1063	18.7	81.4	
Rural	4630	81.3	79.2	
**Age**				3.3 (0.777)
15–19	409	7.2	80.1	
20–24	1275	22.4	79.5	
25–29	1543	27.1	80.7	
30–34	1194	21.0	77.7	
35–39	775	13.6	81.0	
40–44	386	6.8	77.4	
45–49	111	1.9	82.6	
**Marital status**				37.5 (<0.001)
Married	5157	90.6	80.7	
Cohabiting	536	9.4	69.1	
**Health care decision making capacity**				0.7 (0.418)
Alone	431	7.6	75.9	
Not alone	5262	92.4	79.9	
**Parity**				6.1 (0.107)
One birth	430	7.5	76.2	
Two births	699	12.3	76.6	
Three births	830	14.6	82.4	
Four or more births	3734	65.6	80.0	
**Employment status**				2.0 (0.155)
Not working	2622	46.1	79.6	
Working	3071	53.9	79.6	
**Religion**				20.6 (<0.001)
Islam	3058	53.7	81.3	
Christianity	2445	42.9	78.0	
No religion	190	3.4	74.3	
**Frequency of reading newspaper**				6.0 (0.050)
Not at all	5403	94.9	79.4	
Less than once a week	180	3.2	79.8	
At least once a week	110	1.9	89.6	
**Frequency of listening to radio**				12.5 (0.002)
Not at all	4234	74.4	78.0	
Less than once a week	794	13.9	84.9	
At least once a week	665	11.7	83.8	
**Frequency of watching television**				2.7 (0.258)
Not at all	5039	88.5	79.3	
Less than once a week	295	5.2	79.6	
At least once a week	359	6.3	83.6	
**Sex of household head**				1.6 (0.211)
Male	4966	87.2	79.7	
Female	727	12.8	79.0	
**Size of child at birth**				14.2 (0.001)
Larger than average	2615	45.9	77.5	
Average	1644	28.9	83.1	
Smaller than average	1434	25.2	79.5	
**Birth order**				5.4 (0.066)
First	776	13.6	81.0	
2–4	2371	41.7	80.7	
5+	2547	44.7	78.2	
**Twin status**				2.3 (0.132)
Single birth	5531	97.1	79.9	
Multiple births	162	2.9	70.6	
**Sex of child**				0.3 (0.563)
Male	2928	51.4	80.3	
Female	2765	48.6	78.9	

Source: 2014–15 Chad Demographic and Health Survey

**Table 2:** Multilevel logistic regression results on the predictors of healthcare seeking for childhood illnesses in Chad.

Variables	Model 0	Model I	Model II	Model III
		aOR (95% CI)	aOR (95% CI)	aOR (95% CI)
**Getting money for medical care for self**				
Big problem		0.79* (0.64–0.97)		0.80* (0.65–0.99)
Not a big problem		1		1
**Distance to facility for medical care for self**				
Big problem		0.78* (0.65–0.94)		0.79* (0.65–0.95)
Not a big problem		1		1
**Marital status**				
Married		1		1
Cohabiting		0.62** (0.46–0.83)		0.62** (0.47–0.83)
**Religion**				
Islam		1		1
Christianity		0.79 (0.64–0.98)		0.85 (0.66–1.10)
No religion		0.57 (0.10–3.25)		0.60 (0.11–3.43)
At least once a week		0.64 (0.39–1.05)		0.68 (0.41–1.12)
**Frequency of listening to radio**				
Not at all		0.69** (0.54–0.89)		0.71** (0.55–0.91)
Less than once a week		1		1
At least once a week		0.91 (0.65–1.27)		0.87 (0.62–1.22)
**Size of child at birth**				
Larger than average		0.80* (0.67–0.96)		0.79* (0.66–0.95)
Average		1		1
Smaller than average		0.88 (0.71–1.07)		0.88 (0.71–1.08)
**Community literacy level**				
Low			1.06 (0.82–1.37)	0.99 (0.74–1.33)
Medium			0.73* (0.53–0.99)	0.73* (0.53–0.99)
High			1	1
**Community socio-economic status**				
Low			0.68*** (0.53–0.87)	0.82 (0.63–1.07)
Medium			0.83 (0.51–1.34)	0.96 (0.60–1.55)
High			1	1
**Random effect result**				
PSU variance (95% CI)	0.84 (0.64–1.10)	0.73 (0.55–0.98)	0.78 (0.58–1.03)	0.70 (0.52–0.95)
ICC	20%	18%	19%	18%
LR Test	χ2 = 182.89, p<0.001	χ2 = 139.17, p<0.001	χ2 = 162.54, p<0.001	χ2 = 128.83, p<0.001
Wald chi-square		54.13	18.04	62.79
Model fitness				
Log-likelihood	-2684.34	-2657.10	-2675.42	-2652.88
AIC	5372.67	5337.99	5362.83	5297.167
N	5693	5693	5693	5693

Source: 2014–15 Chad Demographic and Health Survey

PSU = Primary sampling unit; ICC = Intra-Class Correlation; LR Test = Likelihood ratio Test; AIC = Akaike’s Information Criterion; N = Sample size

Model 0 is the null model, a baseline model without any independent variable

Model 1 is adjusted for individual level variables

Model 2 is adjusted for community level variables

Model 3 is the final model adjusted for individual and community level variables

### Model fit and specifications

We assessed the fitness of the models with the likelihood ratio (LR) test. The presence of multicollinearity between the independent variables was checked before fitting the models. The variance inflation factor (VIF) test revealed the absence of high multicollinearity between the variables (Mean VIF = 1.21, Max VIF = 1.43, Minimum = 1.05). In order to develop robust models, only variables that showed statistically significant association in the bivariate analysis were included in the models.

### Ethical approval

This study used publicly available data from DHS. Informed consent was obtained from all participants prior to the survey. The DHS Program adheres to ethical standards for protecting the privacy of respondents. The ICF International also ensures that the survey processes conform to the ethical requirements of the U.S. Department of Health and Human Services. No additional ethical approval was required, as the data is secondary and available to the general public. However, to have access and use the raw data, we sought and obtained permission from MEASURE DHS. Details of the ethical standards are available on http://goo.gl/ny8T6X.

## Results

### Healthcare seeking behavior for childhood illnesses in Chad across independent variables

Out of the 17,719 mothers interviewed, 5, 693 reported that their children under five had either fever accompanied by cough or diarrhea or both within the two weeks preceding the survey. Of the 5,693 mothers, 4,592 (79.6%) sought for healthcare for childhood illnesses. Healthcare seeking for childhood illnesses was significantly higher among mothers who cited not a big problem for getting money for their own medical care, distance to a health facility for medical care and wanting to go for medical care alone compared to those who considered these as a big problem. Healthcare seeking for childhood illnesses was high among mothers aged 45–49, the married, those who were not deciding on their healthcare alone, those with three births, those who were working or not, practitioners of Islam and those who read a newspaper at least once a week. Concerning radio, most mothers who listened to radio less than once a week sought healthcare for childhood illnesses, and this also applies to those who watched television at least once a week. Nearly eighty percent of mothers from male-headed and 83.1% of those whose children had average weight at birth sought for healthcare for childhood illnesses. Healthcare seeking dominated for first birth order children, male children, and those having single birth order (Table 1).

With the community level factors, healthcare seeking for childhood illnesses was also more prevalent among mothers with high literacy at the community level (82.0%), those with high socio-economic status at the community level (83.7%) and urban residents (81.4%).

### Multilevel logistic regression results on the predictors of healthcare seeking for childhood illnesses in Chad

Results for all the models were presented in Table 2. In terms of the individual level factors, mothers who faced financial barriers to healthcare access were less likely to seek healthcare for childhood illnesses, relative to those who faced no financial barriers (aOR = 0.80, 95% CI = 0.65–0.99). Mothers who reported that distance to the health facility was a barrier were less likely to seek healthcare for childhood illnesses compared to those who faced no geographical barrier to healthcare access (aOR = 79, 95% CI = 0.65–0.95). Mothers who were cohabiting were less probable to seek healthcare for childhood illnesses (aOR = 0.62 95% CI = 0.47–0.83) compared to married mothers (aOR = 0.62 95% CI = 0.47–0.83). Lower odds of childhood healthcare seeking was noted among mothers who did not listen to radio at all, relative to those who listened to radio at least once a week (aOR = 0.71, 95% CI = 0.55–0.91). Mothers whose children were larger than average size at birth had a lesser likelihood of seeking childhood healthcare, compared to those whose children were of average size (aOR = 0.79, 95% CI = 0.66–0.95).

We further noted that with the community level factors, mothers who lived in communities with medium literacy level were less likely to seek childhood healthcare than those in communities with high literacy (aOR = 0.73, 95% CI = 0.53–0.99) (Table 2, Model III).

## Discussion

In the present study, we found that individual (financial barriers to healthcare access, geographical barriers to healthcare access, marital status, frequency of listening to radio, and size of children at birth) and community level factors (community level literacy) are associated with healthcare seeking behaviour for childhood illnesses.

Findings on the association between financial barriers and healthcare seeking for childhood illnesses support the findings of previous studies in Kenya, Nigeria and Niger [20] Ethiopia [29] and sub-Saharan Africa [30, 31]. The possible reason for the finding could be that mothers who face financial barriers may not be able to pay for the cost of healthcare and this is likely to inhibit them from seeking healthcare for their children. Closely related to financial barriers are geographical barriers, due to long distance to health facilities, which also put financial burdens on mothers [32].

Consistent with some previous studies, the present study revealed a statistically significant association between mass media exposure and health seeking behaviour for childhood diseases [33, 34]. In explaining this association, Gebretsadik et al. [34] and Adinan et al. [33] noted that mass media are sometimes used to create awareness on health issues; therefore, mothers’ access to mass media increases their awareness of the importance of child healthcare, and this may explain their higher likelihood of seeking healthcare for their sick children. Exposure to media may also be associated with wealth and reduction in poverty, which can also facilitate healthcare seeking.

The study also reveals a significant association between the marital status of mothers and healthcare seeking behaviour of childhood diseases. A study in Nigeria similarly reported a significant association between maternal marital status and healthcare seeking behaviour for childhood illnesses [35]. It has been explained that marriage is associated with better health [34]. Moreover, marriage offers emotional connectedness or support [36], which may encourage one to behave in a manner that promotes health. A sense of responsibility toward a spouse or encouragement from that spouse may push men and women to behave in ways that prevent health problems or promote better health [36]. In the context of the present study, the high probability of married mothers to seek healthcare for their children’s illnesses may be due to the financial and emotional support they receive from their husbands during pregnancy and childbirth.

Finding on the association between community literacy level and healthcare seeking for childhood illnesses concurs with the findings of studies carried out in sub-Saharan Africa [37, 38] and Ethiopia [39], where low community literacy among mothers was found to reduce healthcare seeking for childhood illnesses. The possible reason for the finding is that mothers who live in communities with high literacy levels are more likely to have access to all kinds of information on the need to seek care for their children’s diseases. Access to such information could encourage them to seek care for their children’s illnesses. More, mothers living in more literate communities are themselves more likely to be literate.

Mothers who had the perception that their children were larger than average at birth were less likely to seek healthcare for childhood illnesses compared to those whose children had average size at birth. Similar findings have been obtained in previous studies in sub-Saharan Africa [38, 40]. The authors are unable to provide reasons for this finding and consider this finding as a gap that should be explored in future studies since the size of the child at birth was obtained from the subjective opinion of mothers.

## Strengths and limitations

The main strength of the study is its use of nationally representative data. Besides, the data collection for the survey featured standard data collection methods, including the use of experienced researchers. This resulted in a high response rate. Moreover, in the study, we used higher-order statistical analysis tools for the analysis. In terms of limitations, it must be acknowledged that the cross-sectional study design adopted prevents us from drawing causal relations between the variables studied. Also, the use of secondary data limited our analyses to only the variables identified in the dataset and excluded variables that were not in the dataset used. Finally, the use of mothers’ perception of the size of the child at birth as an explanatory variable is a limitation of this study since their responses may be prone to bias.

## Conclusion

The study revealed that both individual (financial barriers to healthcare access, geographical barriers to healthcare access, marital status, frequency of listening to radio and size of children at birth) and community level factors (community level literacy) are associated with healthcare-seeking behaviour for childhood illnesses in Chad. The government of Chad, through multi-sectoral partnership, should strengthen health systems by removing financial and geographical barriers to healthcare access. Moreover, the government should create favourable conditions to improve the status of mothers and foster their overall socio-economic wellbeing and literacy through employment and education. Other interventions should include community sensitization of cohabiting mothers and mothers with children whose size at birth is large to seek healthcare for their children when they are ill. This can be done using radio as means of information dissemination.

## Abbreviations

AOR: Adjusted Odds Ratio
CI: Confidence Interval
DHS: Demographic and Health Surveys
CDHS: Chad Demographic and Health Survey
WHO: World Health Organization
SDG: Sustainable Development Goal
SSA: sub-Saharan Africa
VIF: Variance Inflation Factor
LR: Likelihood Ratio
